# Young Offspring at Genetic Risk of Adult Psychoses: The Form of the Trajectory of IQ or Memory May Orient to the Right Dysfunction at the Right Time

**DOI:** 10.1371/journal.pone.0019153

**Published:** 2011-04-29

**Authors:** Michel Maziade, Nancie Rouleau, Caroline Cellard, Marco Battaglia, Thomas Paccalet, Isabel Moreau, Valérie Gagnon, Nathalie Gingras, Cecilia Marino, Elsa Gilbert, Marc-André Roy, Chantal Mérette

**Affiliations:** 1 Laval University and Centre de Recherche Université Laval Robert-Giffard, Laval, Québec, Canada; 2 Faculté de Médecine, Université Laval, Laval, Québec, Canada; 3 École de psychologie, Université Laval, Laval, Québec, Canada; 4 Academic Centre for the Study of Behavioural Plasticity, Vita-Salute San Raffaele University, Milan, Italy; 5 Department of Child Psychiatry, Eugenio Medea Institute, Bosisio Parini (Lecco), Italy; Institute of Automation, Chinese Academy of Sciences, China

## Abstract

**Objective:**

Neurocognitive dysfunctions analogous to those of adult patients have been detected in children at risk of schizophrenia and bipolar disorder. This led to the following developmental question: Do IQ and memory impairments exhibit different developmental courses from childhood to young adulthood in terms of stability or fluctuations?

**Methods:**

In a high risk sample, we used a step by step sampling approach to narrow-down the early disease mechanisms. Upstream, we started with a 20-year follow-up of 48 densely affected multigenerational kindreds, including 1500 clinically characterized adult members. We then identified 400 adult members affected by a DSM-IV schizophrenia or bipolar disorder. Downstream, we finally focused on 65 offspring (of an affected parent) aged 7 to 22, who were administered a neuropsychological battery. We then constructed cross-sectional trajectories that were compared to those of controls.

**Results:**

The childhood IQ deficit displayed a stability until young adulthood. The delay in visual memory exhibited a non-linear two-stage trajectory: a lagging period during childhood followed by a recuperation period from adolescence until adulthood, as supported by a significant *Group x Age Periods* interaction. No data suggested deterioration between 7 and 22.

**Conclusion:**

In these offspring at genetic risk, the developmental trajectory of global IQ impairment may not apply to specific domains of cognition such as episodic memory. Different cognitive dysfunctions would mark different developmental courses. The shape of the trajectories might itself have a meaning and provide empirical leads for targeting the right dysfunction at the right time in future prevention research.

## Introduction

Neuropsychological deficits have been documented in schizophrenia and may be at the core of pathological processes. Patients with schizophrenia have pronounced impairments in general intelligence level (IQ), verbal memory and processing speed, with large effect sizes of 1.0 and higher [1]–[7]. Bipolar patients present the same deficits but less severely [4]. Cognitive deficits precede the appearance of adult disease [8] and children or adolescents (from 3 to 21 years old) who develop schizophrenia usually present a deficit of 5–9 points in global IQ [9]–[12]. Many studies confirmed a further decline of 5–8 points of IQ in adult schizophrenic patients who have a IQ impairment that is around twice as large as that in childhood [13], [14]. However, little is known about the time incidence of such a “second drop” in IQ among vulnerable individuals. Most of the longitudinal studies on cognitive impairments were centered on IQ [15], [16] and few studies measured other cognitive domains such as memory [17]–[19].

Although an increasing number of studies focused on detecting high-risk individuals by means of cognition in the prodromal years (late teen or early twenties), few studies in developmental psychopathology investigated the long-term course of cognitive dysfunctions in birth cohorts or in high-risk offspring of an affected parent [11], [15], [16], [20]–[22], and most of the latter had their cognitive measures taken only between 3 and 13 years of age. This leaves a void of developmental information on how dysfunctions evolve from childhood, to adolescence and into early adulthood. In the children of the Dunedin birth cohort who had developed schizophreniform disorder at age 26, Cannon et al [15] reported a fairly stable deficit of global intelligence level (IQ) between 3 to 9 years of age along with motor and language delays. In the same cohort, Reichenberg et al [16] used the subtests of the Weschler intelligence scale (IQ) measured between age 7 and 13 and uncovered two distinct trajectories related to different cognitive clusters predicting psychosis at age 32. Erlenmeyer et al's [18] long-term follow-up of 9 year-old offspring of a schizophrenic parent found delays in diverse developmental milestones. A combination of verbal memory, attention and motor delays distinguished the children who later developed the illness.

In sum, longitudinal studies reveal an early IQ impairment that is stable from childhood until late adolescence and other developmental delays in the children who will develop major psychoses. However, most studies ended with a small number of individuals affected at adult outcome and they focused on the childhood years with a limited array of cognitive functions, yielding little knowledge on episodic memory, particularly visual memory, and on how developmental trajectories of different cognitive dysfunctions may relate to the onset of psychoses.

The follow-up of birth cohorts or of children at genetic risk take a long time to yield results. We thus combined *developmental psychopathology* and *family genetics* into an approach using cross-sectional assessments of three complementary sub-samples (adult patients, non-affected adult relatives and young offspring of an affected parent) from our Eastern Quebec kindreds densely affected by schizophrenia or bipolar disorder [23], [24]. We paid particular attention to episodic memory for three empirical reasons. First, using an extended neuropsychological battery, we found that verbal and visual episodic memory were among the most affected functions in adult patients [25]. We also observed that the non-affected adult relatives of these patients displayed verbal episodic memory deficits [25]. The findings in these kindreds were consistent with the observations made in less familial or general samples of schizophrenia or bipolar patients [1]–[4] and their non-affected relatives [1], [5], [26]–[28]. Second, episodic memory also yielded the largest effect sizes [8] in our sample of young offspring at genetic risk, suggesting memory dysfunctions a long time before the prodrome and onset of illness, which is compatible with the literature [17], [18], [29]–[31]. Third, we found different inter-generational predictive patterns for visual and verbal memory impairments [25]. The visual memory dysfunction present in the offspring was also observable in the adults who converted to major psychoses, but not in the adult relatives who did not. This suggested that visual memory fitted a more specific disease precursor model than verbal memory, a finding compatible with Skelley et al's study [5] showing that visual memory impairments were present in schizophrenic patients but absent in their non-affected adult relatives. It must be noted that visual episodic memory has so far been modestly investigated in major psychosis in comparison to verbal memory and little knowledge exists about their developmental trajectories.

Trajectories in developmental disorders may be constructed using three methods, each having advantages and disadvantages [32]: i) on data collected on a single point in time in a cross-sectional sample varying in age; ii) on data colleted in multiple points in time in individuals of the same age or iii) in a combination of both methods. The wide age range (7 to 22) of our sample of offspring made the first method particularly suitable. Cross-sectional data can be used to outline trajectories provided that special biases and false inferences are avoided [33]. For instance, using the mean and variance at each of the different times studied is warranted [33].

By means of cross-sectional trajectories studied in offspring at high genetic risk, our objective herein was to look at the stability or the variations of the IQ and episodic memory impairments in four different age periods from the primary school years through adolescence up to adulthood.

## Methods

### Sample

**Figure 1:**
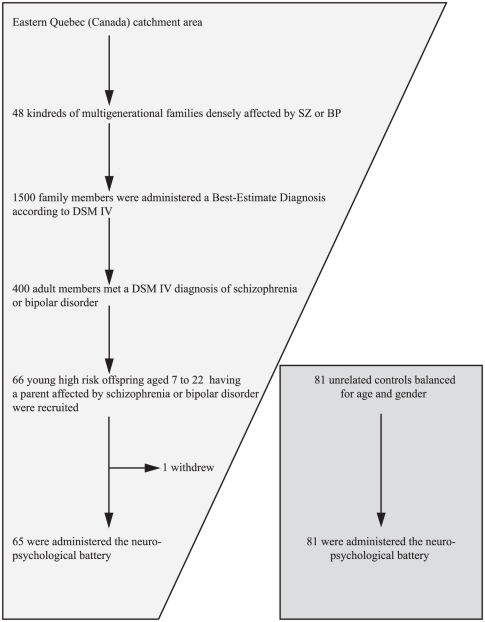
Flowchart of participants in a stepwise sampling approach to narrow-down the early disease mechanisms in this high-risk sample. In a high risk sample, we used a step by step sampling approach to narrow-down the early disease mechanisms. Upstream, we started with a 20-year follow-up of 48 densely affected multigenerational kindreds, including 1500 clinically characterized adult members who were all administered a consensus best-estimate lifetime diagnosis. From these, we then identified 400 members affected by a DSM-IV schizophrenia or bipolar disorder. Downstream, we finally focused on 65 offspring aged 7 to 22 having an affected parent, and the 65 were administered the neuropsychological battery.

Our stepwise selection strategy in this high-risk sample is detailed in **Figure 1**.

#### Ascertainment of kindreds

All the multigenerational families densely affected by schizophrenia or bipolar disorder in the Eastern Québec (Canada) catchment area were targeted. Family *inclusion criteria* were: i) having at least one first-degree relative affected with the same disorder (schizophrenia or bipolar disorder) as the proband; ii) having at least four affected individuals sharing the same disorder. We gathered 48 schizophrenic or bipolar kindreds over 20 years with an average of 26 members per kindred including an average of six affected by schizophrenia or bipolar disorder. The mean age of disease onset was 25.4 (SD 8.5) years for schizophrenia and 28.8 (SD 10.3) years for bipolar disorder. More details about the kindred ascertainment are available elsewhere [23], [24], [34]–[36].

#### Young offspring and control samples

The sample description is in **Table 1**. As previously described [25], the high risk offspring *inclusion criteria* were: having a parent with a DSM-IV schizophrenia or bipolar disorder, and being aged below 23. The offspring *exclusion criteria* were: the presence of a diagnosis of DSM-IV psychotic disorder, bipolar disorder or major depression, and brain and metabolic disorders known to cause neuropsychological impairments. Compared to our former report [25], 5 new recruitments increased the sample from 60 to 65 subjects. As observed in other samples [19], [37], [38], we previously reported that a proportion of these offspring had a non-psychotic DSM diagnosis [39] and that the presence of such diagnoses did not affect their cognitive differences with controls [8]. There was no difference in socioeconomic status between the total samples of offspring (*N* = 65) and controls (*N* = 81) (see **Table 1**<\emph>). The offspring sample included a number of sibships and this was taken into account in our statistical analyses.

**Table 1:** Sociodemographic description of the samples.

	Total sample		
Sociodemographic variables	Offspring	Controls	*p-values* 1
	(n = 65)	(n = 81)	
Mean age at cognitive evaluation (SD)	17.1 (4.2)	17.1 (4.0)	.91 (NS^2^)
Age range	(7.5–22.9)	(7.7–22.9)	
Number of males (%)	34 (52)	40 (49.4)	.73 (NS)
Socioeconomic status (SD)^3^	39.5 (15.6)	44.7 (18.3)	.08 (NS)
	Min: 22.08	Min: 22.08	
	Max: 70.19	Max: 75.87	

1 *p-values* obtained from *t* test for age and socioeconomic status and from χ^2^ test for the number of males.

2 NS: non significant *p-value*.

3 We used the Blishen index [56] according to the highest socioeconomic status of the two parents. This index is based on education and income and on a Canadian census of 514 occupational categories according to the Canadian Classification and Dictionary of Occupations. Offspring and controls were not different on socioeconomic status. When the socioeconomic status was entered as a covariate, the difference between offspring and controls remained the same on the neuropsychological measures: Global IQ (p<.001), CVLT_TOT_ (p<.001), CVLT_DR_ (p < .001), RCFT_IR_ (p<.001) and RCFT_DR_ (p<.001).

Healthy unrelated controls balanced for age and gender were recruited concurrently with offspring through advertisements from the same population. *The exclusion criteria* were the same as those for offspring. In addition, controls had no lifetime axis I DSM diagnosis and no positive family history of schizophrenia or bipolar disorder spectrum disorders.

We divided the offspring into developmental age periods corresponding to childhood (7 to 12 years of age; *N* = 13), early-adolescence (13–16; *N* = 15), late-adolescence (17–19; *N* = 15) and young adulthood (20–22; *N* = 22). The normal control group was similarly divided: *N* respectively of 14, 28, 14 and 25. Comparable developmental age periods were used in former studies and also in the interpretation of a meta-analysis of 18 studies [9].

The study was approved by the University Ethical Committee of the Quebec Mental Health Institute (Laval University, Quebec, Canada). The study was personally explained and all participants gave written informed consent; for minors, written informed consent was obtained from the parents/guardians.

### Measurements

#### Psychiatric ascertainment of parents and offspring

A best estimate lifetime diagnostic procedure was administered to the parents, their adult relatives and the offspring [24], [39]. This best-estimate procedure reviewed all available medical records, family informant interviews and the semi-structured interview. The Kiddie-Schedule for Affective Disorders and Schizophrenia (K-SADS) [40] was administered with the parents of children under 18 in the presence of the child, and the Structured Clinical Interview for DSM disorders (SCID) [41] to the subjects over 18. The healthy controls were assessed with the K-SADS or the SCID to exclude axis I or II diagnosis.

#### Neuropsychological assessments

In the present study, we report on the cognitive differences by age periods on IQ and on the free recall measures of verbal episodic memory and visual episodic memory. IQ was assessed with a full standard intelligence scale (Wechsler Intelligence Scale for Children, WISC-III, or Wechsler Adult Intelligence Scale, WAIS-III, after 16 years) [42]–[44]. Verbal episodic memory was measured with the *California Verbal Learning* Test (CVLT). Participants had to learn a series of words presented orally over 5 trials and to recall them after each presentation (total recall of 5 trials, CVLT_TOT_) and with a 20-minute delay (delayed recall, CVLT_DR_). Visual episodic memory was assessed with the *Rey Complex Figure* Test (RCFT) [45]. Participants had to copy a complex figure and then recall it from memory after 3 minutes (immediate recall, RCFT_IR_) and 30 minutes (delayed recall, RCFT_DR_).

### Statistical Analysis

IQ and episodic memory functioning in our high-risk offspring has already been reported as significantly lower than normal controls [8], [25]. To further characterize these offspring, the aim of the present study was to inspect the stability or the variations of IQ and memory impairments across four age periods from 7 to 22, by constructing cross-sectional trajectories. To do so, we compared the HR offspring and controls divided into four groups of different age. We performed an analysis of covariance (ANCOVA; SAS version 9.2) on the scores of the cognitive tests, i.e. standardized scores for global IQ and raw scores for verbal episodic memory (CVLT_TOT_, CVLT_DR_) and visual episodic memory (RCFT_IR_, RCFT_DR_). Gender was selected as a covariable in all statistical analyses. To account for the non-independance of observations within the same sibship, a multilevel regression analysis was applied with the MIXED procedure of SAS (version 9.2). Sibships nested in the group were used as the second level and modeled according to a random effect. Degrees of freedom were obtained by the method of Kenward-Roger [46], available with the option DDFM  =  KR in the MODEL statement of the MIXED procedure. ES were calculated using the difference of adjusted means (LSMeans) between the experimental and control groups standardized by a pooled standard deviation. The pooled standard deviation was obtained by dividing the standard error of the difference of LSMeans by the square root of 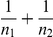 [47]. Confidence intervals (CIs) for the effect sizes were obtained using the non-centrality interval estimation approach based on a ‘t’ distribution [48]. The lower and upper bounds of the 95% CI were calculated by multiplying the 2.5% and 97.5% percentiles, respectively, of the non central ‘t’ distribution by the square root of 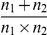.

To test whether or not the cognitive differences between offspring and controls differed across the age periods, we computed a *Group x Age Periods* interaction term. When an interaction was detected at *p* = .05, *multiple testing* for the main effect was accounted for by setting the threshold for significance at *p* = .01 (i.e. the usual .05 divided by the number of different cognitive measures).

Using the UNIVARIATE procedure of SAS, we verified for all the cognitive variables that the Skewness and the Kurtosis coefficients were between −1 and 1 and required that all three tests of normality of the residuals provided by default by SAS, in addition to the Shapiro-Wilk one (option NORMAL), were non significant.

## Results

Congruent with our previous reports [8], [25], the offspring performance was found lower than that of controls for IQ and episodic memory. The main effect of *Groups* (offspring vs controls) was found significant for Global IQ (*p*<.0001), Verbal memory (CVLT_TOT_, *p*<.0001; CVLT_RD_, *p*<.0001) and Visual memory (RCFT_IR_, *p*<.0001; RCFT_DR_, *p*<.0001) (**Table 2**).

**Table 2:** Comparisons of offspring to controls in the total samples and in subsamples of different age periods.

Neuropsychological Variables																				
	Global IQ				CVLT_TOT_				CVLT_DR_				RCFT_IR_				RCFT_DR_			
Source	*df*	F	*p*	ES*(CI)*	*df*	F	*p*	ES*(CI)*	*df*	F	*p*	ES*(CI)*	*df*	F	*p*	ES*(CI)*	*df*	F	*p*	ES*(CI)*
Groups^1^	1	25.58	<.0001	−0.84*(*−*1.18,* −*0.5)*	1	25.41	<.0001	−0.85*(*−*1.2,* −*0.49)*	1	31.99	<.0001	−0.95*(*−*1.30,* −*0.59)*	1	20.36	<.0001		1	23.58	<.0001	
Age periods	3	1.33	0.2685		3	5.56	0.001		3	2.93	0.036		3	5.25	0.002		3	5.77	0.001	
Groups X Age periods	3	0.45	0.7162		3	1.46	0.23		3	2.58	0.056		3	2.74	0.047		3	2.96	0.035	
Simple main effects																				
Offspring vs Controls at 7–12 years														−1.68	0.096	−0.66*(–1.19, 0.35)*		–1.32	0.19	–0.52*(–1.29, 0.25)*
Offspring vs Controls at 13–16 years														–4.75	<.0001	–1.52*(*–*2.17,* –*0.86)*		–4.78	<.0001	–1.53*(*–*2.18,* –*0.87)*
Offspring vs Controls at 17–19 years														–2.09	0.038	–0.79*(*–*1.54,* –*0.04)*		–3.06	0.003	–1.16–*1.91,* –*0.4)*
Offspring vs Controls at 20–22 years														–1.11	0.27	–0.32*(*–*0.9, 0.25)*		–1.37	0.17	–0.4*(*–*0.97, 0.18)*

1 Comparison of total sample of offspring to total sample of controls.

*– Non-independence of observations:* The 65 offspring sample was composed of 26 singletons and 17 sibships: 12 comprised two subjects and 5 comprised three. To account for possible correlation among subjects within the same sibship, a multilevel model was carried out using the MIXED procedure of SAS (version 9.2; SAS Institute Inc., Cary, NC). Sibships nested in the group were used as the second level and modeled according to a random effect. Degrees of freedom were obtained by the method of Kenward-Roger, 1997 [46] that is available with the option DDFM = KR in the MODEL statement of the MIXED procedure.

*Effect sizes (ES):* ES were calculated using the difference of adjusted means (LSMeans) between the experimental and control groups standardized by a pooled standard deviation. The pooled standard deviation was obtained by dividing the standard error of the difference of LSMeans by the square root of 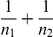, according to Kelley [47]. Confidence intervals (CI) for the effect sizes were obtained using the non-centrality interval estimation approach based on a *t* distribution, according to Steiger and Fouladi, 1997 [48]. The lower and upper bounds of the 95% CI were calculated by multiplying the 2.5% and 97.5% percentiles, respectively, of the non central *t* distribution by the square root of 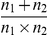.

*– Interaction term:* A statistically significant interaction term (*Group x Age Periods*), as described in *Statistical Analysis,* was found only for the two *Rey Complex Figures* Tests (immediate recall and delayed recall, respectively RCFT_IR_ and RCFT_DR_) suggesting that the cognitive differences for this domain varied with age. This imposed to interpret the effect sizes at each age period rather than the marginal one.

**Figure 2:**
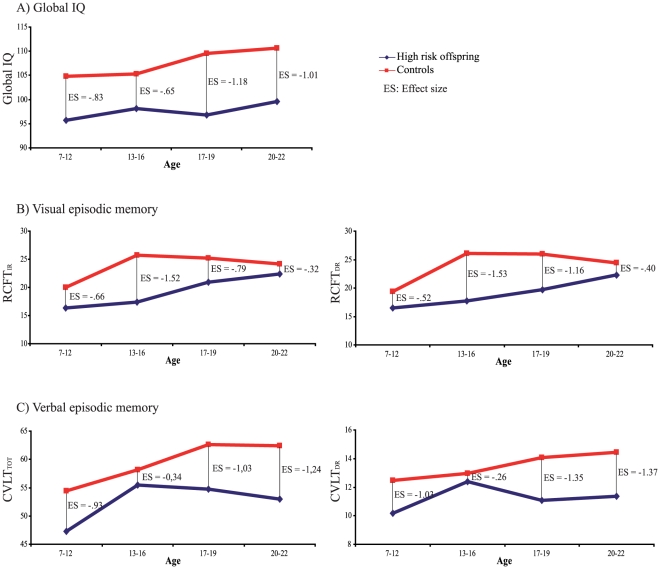
Cross-sectional developmental trajectories representing the evolution in time of the IQ and memory impairments from 7 to 22 years of age. The developmental pattern for Global IQ (A), Visual episodic memory (B) and Verbal episodic memory (C) are illustrated for the controls (red line) and young offspring at genetic risk (blue line). The effect sizes (ES) express the magnitude of the difference between offspring and controls at each age period. The statistically significant *Group x Age Periods* interaction term obtained only for the two tests of visual episodic memory (*p*  =  .047 for Rey Complex Figure Test immediate recall, RCFT_IR_, and *p*  =  .035 for Rey Complex Figure Test immediate recall, RCFT_DR_) suggested that the cognitive differences varied with age for this function. A trend approaching significance could be detected for one of the two tests of verbal episodic memory (CVLT_DR_, *p*  =  .056).

The *Group x Age Periods* interaction term reached statistical significance only for the two tests of visual memory (RCFT_IR_, *p* = .047; RCFT_DR_, *p* = .035; **Table 2**) suggesting different degrees of impairment at the different age periods. We then decomposed the latter interaction through post-hoc *t* tests (**Table 2** **;** **Figure 2**). A first observation was a moderate delay at 7–12 years for the two tests (Effect Size; ES = −.66 and −.52 respectively for RCFT_IR_ and RCFT_DR_) which tended to increase in young adolescence (ES = −1.52 and −1.53). The second observation for visual memory was that the difference between groups decreased from their peak during adolescence to young adulthood for the two tests (young adulthood ES respectively of −.32 and −.40 for RCFT_IR_ and RCFT_DR_) ending closer to normal controls. In contrast, global IQ displayed a stable deficit of around 7–11 points from the primary school years until adulthood (**Figure 2** **)**. As regards verbal episodic memory, a possible trend of interaction present in only one of the two tests (CVLT_DR_, *p* = .056; CVLT_IR_, *p* = .23 NS) provided inconclusive results. None of the examined cognitive functions exhibited a deteriorating evolution, i.e. normality in childhood followed by a decline of performance.

To verify the possibility that the results might depend on the chosen developmental age cut-offs, we re-examined the developmental patterns with three age periods (ages 7–14, 15–18, 19–22) and the results were congruent (**Table S1** *in Supporting information S1*). Indeed, the *Group* variable was significant for all the cognitive functions tested. As in the “four period analysis”, there were no statistically significant *Group x Age Periods* interaction terms for the Global IQ and for the verbal memory tests. In contrast, for the two tests of visual memory, the interaction term showed a statistical trend (RCFT_IR_, *p* = .071; RCFT_DR_, *p* = .099; **Table S1**) with shapes of the trajectories similar to those of the four age periods i.e. a lagging period during childhood followed by a recuperation period from adolescence until adulthood (**Figure S1** *in Supporting information S1*), whereas Global IQ continued to display a stable deficit.

## Discussion

To our knowledge, this study is the first to provide comparative information on developmental trajectories of IQ and episodic memory impairments using the same measures across early childhood, adolescence and young adulthood in offspring at genetic risk of major psychoses. We had formerly reported sizable cognitive deficits in these offspring [8], [25] and we now observe three developmental patterns in this high risk sample that may be of high relevance for prevention research: i) the detection in early childhood of a lowered global IQ that remained stable until age 22; ii) the difference between IQ and memory impairments in their long-term developmental course and iii) a two-stage developmental course for visual memory characterized by an initial childhood period of slowed development and followed by a “recuperation” trend until young adulthood.

### Stability over time of the developmental delay in general intelligence

**Figure 3:**
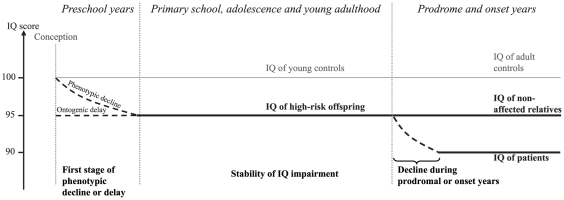
Illustration of a putative three-stage progression toward disease for Global IQ impairment. The first stage would extend from conception/pregnancy until 3 to 6 years old and would be characterized by a phenotypic decline of 5–8 points and/or ontogenetic delay in children at risk. During the second stage, from primary school years until young adulthood, youths at risk would present a stable IQ deficit. The third stage, beginning around the prodromal years, illustrates the drop of 5–8 points of IQ suffered by patients. Dotted lines represent sequences of change or decline periods during the life of an individual, plain lines represent stability period in IQ. This study encompassed only the period of *Primary school, adolescence and young adulthood*.

We observed that the global IQ deficits previously reported in these offspring [8], [25] was steadily present from entry to primary school until young adulthood, supporting the neurodevelopmental etiology for major psychosis [49]. This stability in IQ impairment is congruent with former studies of children who later developed schizophrenia and with the conclusion of a meta-analysis of 18 studies using developmental age periods resembling our own [9]. Adult patients from our kindreds [25] and from other cohorts [13], [14] generally present an IQ deficit at least twice as large as that in high-risk children. These levels of general intelligence impairments in the premorbid period compared to those seen in patients might imply a three-stage progression toward disease **(** **Figure 3**). First, an IQ delay installed before school age, second, a stable impairment that follows until age 20–22, and a third stage of further decline taking place during the prodromal/early onset years. Importantly, the developmental course of global IQ would not necessarily apply to specific domains of cognition as discussed below.

### Evolution over time of the verbal and visual memory delays

In contrast to the stable course of the IQ impairment from age 7 to 22, our data suggest for visual memory a two-stage developmental course. There would be an initial slowed development during childhood marked by a lag (or increasing distance from the functioning of controls) until young adolescence. This would be followed by a “recuperation” period from adolescence to young adulthood when the offspring memory performance would end closer to that of controls. This pattern is supported by a significant *Group x Age Periods* interaction for the two tests of visual memory suggesting that the cognitive difference with controls varied across time. When the cross-sectional trajectories in **Figure 2** were further inspected, the delays in visual memory were moderate at 7–12 years (ES = −.66 and −.52 respectively for RCFT *immediate recall* and RCFT *delayed recall*), increased in young adolescence (ES = −1.52 and −1.53) and through late adolescence, and ended up closer to controls at age 20–22 (ES of respectively −.32 and −.40).

As regards verbal memory, the magnitude of the delay appeared already large for the two tests early at 7–12 years (ES = −.93 *CVLT immediate recall* and −1.03 *CVLT delayed recall*) and remained similarly large at age 20–22 (respectively ES = −1.01 and −1.37). In the interval, a trend approaching significance for the *Group x Age Periods* interaction, in only one of the two tests of verbal memory (CVLT_DR_), prevented us from concluding there was a possible variation across age-periods.

The present finding of an apparent recuperation trajectory for visual memory should be harmonized with our previous report [25] of impaired visual memory in patients. From a methodological standpoint, we previously demonstrated that our results in visual memory were not due to a bias in our control sample [25] since our controls values were close to the published normative values [45]. From a developmental and genetic point of view, visual episodic memory could be underlain by neural systems that have more plasticity than other cognitive regions. Thus, it appears conceivable that while an impaired visual memory would indicate heightened risk of developing illness, it is the ability to either plastically catch-up or remain stably low in visual memory tests that will discriminate those adolescents who will not progress toward developing psychosis from those who will. Thus, while adolescents with decreased visual memory would constitute a group of – temporarily – unexpressed risk for psychosis, those who fail to show spontaneous catching up at the RCFT (or similar) tests may deserve special attention, as they would be more likely to manifest the symptoms of illness after adolescence.

If these results were replicated, dysfunctions in specific cognitive processes, such as in episodic memory, would exhibit singularities in their developmental course suggesting that each dysfunction may be underlain by its own gene-environment mechanism [25], [50], [51]. Our findings accommodate somewhat those of Reichenberg et al [16] who also observed different trajectories for different cognitive deficits (derived from the WISC subtests) in the children who later developed psychosis. Differences in sampling, in measures and in time period preclude further comparisons between the two studies. The present indices of difference in verbal and visual memory trajectories would also be congruent with two other observations previously reported in our kindred sample: the distinct predictive pathways for visual and for verbal memory in an analysis of the generational differences [25], and the absence of correlation between the verbal and visual memory measures in the offspring sample [8].

### Implications for the design of future risk and prevention research: the importance of timing

This possible catching-up process in the visual episodic memory trajectory calls for further research since it may indicate a corresponding latent capacity for neural plasticity and, for that reason, could emerge as a more appropriate cognitive target for research in preventive remediation. Our data also bring to light the importance of focusing future prevention research on the right cognitive function at the right time in the child's life. Nothing is known as to whether preventive remediation should target cognitive delays that are more marked or more stable across development, or rather target dysfunctions that would show a naturalistic propensity to plasticity and recuperation. In that respect, recent findings in psychiatric disorders suggest compensatory cognitive mechanisms that would overcome genetic or vulnerability dysfunctions [52]. In any case, our data suggest that the developmental courses of specific cognitive functions should be investigated separately in future long-term predictive studies.

### Limitations of the study

One should keep in mind the limitations of our methods. *First*, the obtained developmental courses across age periods are not longitudinal measures taken in a follow-up of offspring. However, data collected on a single point in time in a cross-sectional sample varying in age, here using the same measures in offspring subsamples of different age periods, represent a reliable source of information about trajectories in child developmental disorders [32]. *Second,* the small sample sizes may have generated type 2 errors. *Third,* the present high-risk sample contained carriers and non-carriers of the susceptibility genes, contrary to a longitudinal sample followed until the appearance of the disease. In counterpart, one strength is that these offspring descend from a homogeneous and well characterized kindred from the same population [24], [53] with the unequivocal risk status due to the heavy familial loading. *Fourth*, the generalization of the results obtained in these offspring at high genetic risk may be limited. However, these offspring presented a stability in IQ impairment that is consistent with former studies of birth cohorts or of high-risk offspring of less familial or sporadic parents [9], and their reported rate of non-psychotic DSM disorders [39] was also similar to other samples [19], [38], [54]. Finally, the size of our sample did not permit to accurately compare the developmental courses of schizophrenia and bipolar disorder and our data cannot eliminate the possibility that the quantitative difference in cognitive impairments observed between the two disorders [4] could take origin in differential forms of early trajectories or different sensitive periods. We already reported that the cognitive differences in several domains including episodic memory were shared by the two disorders, either in the patients, their adult non-affected relatives or in the young offspring of an affected parent [8], [25], congruent with other studies of non- or less familial samples [1]–[5].

### Conclusive remarks

In sum, our findings suggest that cognitive deficits are not evanescent and that visual memory delays and general intelligence delays do not follow the same evolution in time. Moreover, the shape of the trajectories itself may provide empirical leads for targeting the right dysfunction at the right time in prevention research.

Finally, our findings point to a heartbreaking human condition. Children and adolescents at genetic risk for major psychoses, not just those descending from multi-affected families, do not start their lives on an equal footing with other children. Cognitive [8], social and behavioural handicaps [39] start burdening them in primary school and thereafter [17]–[19], [54], [55]. What is required is more research on evidence-based and ethical psychosocial, cognitive and pharmacological means of prevention, and means to identify the gene carriers among these children.

## Supporting Information

Supporting Information S1 This information suggests that the results presented in the main text did not depend on the chosen developmental age cut-offs. To allow the construction of a developmental trajectory, the offspring sample was divided into subsamples of different age. Since there is no empirical evidence on which to base the choice of age cut-offs, we chose cut-offs that correspond to developmental ages that may have a meaning in social, clinical and developmental psychopathology. Thus, in the text of our article, a four age-period was chosen, as follow: primary school years (age 7–12), young adolescence (13–16), late adolescence (17–19) and beginning of adulthood (20–22). When we re-examined the developmental patterns with three age periods (ages 7–14, 15–18, 19–22), the results remained congruent (**Table S1**). To further analyze the three period intervals, it can be noted that the *Group* variable was significant for all the cognitive functions tested. As in the “four period analysis”, there were no statistically significant *Group x Age Periods* interaction terms for the Global IQ and for the verbal memory tests. In contrast, for the two tests of visual memory, the interaction term showed a statistical trend (RCFT_IR_, *p* = .071; RCFT_DR_, *p* = .099; **Table S1**) with shapes of the trajectories similar to those of the four age periods i.e. a lagging period during childhood followed by a recuperation period from adolescence until adulthood (**Figure S1**), whereas Global IQ displayed again a stable deficit.
(DOC)
