# Supplementary material for: Young Offspring at Genetic Risk of Adult Psychoses: The Form of the Trajectory of IQ or Memory May Orient to the Right Dysfunction at the Right Time
Source: PLoS One. 2011 Apr 29;6(4):e19153. doi: 10.1371/journal.pone.0019153 (PMC3084759; doi:10.1371/journal.pone.0019153)
Supplement: Supporting Information S1 — This information suggests that the results presented in the main text did not depend on the chosen developmental age cut-offs. To allow the construction of a developmental trajectory, the offspring sample was divided into subsamples of different age. Since there is no empirical evidence on which to base the choice of age cut-offs, we chose cut-offs that correspond to developmental ages that may have a meaning in social, clinical and developmental psychopathology. Thus, in the text of our article, a four age-period was chosen, as follow: primary school years (age 7–12), young adolescence (13–16), late adolescence (17–19) and beginning of adulthood (20–22). When we re-examined the developmental patterns with three age periods (ages 7–14, 15–18, 19–22), the results remained congruent (Table S1). To further analyze the three period intervals, it can be noted that the Group variable was significant for all the cognitive functions tested. As in the “four period analysis”, there were no statistically significant Group x Age Periods interaction terms for the Global IQ and for the verbal memory tests. In contrast, for the two tests of visual memory, the interaction term showed a statistical trend (RCFTIR, p = .071; RCFTDR, p = .099; Table S1) with shapes of the trajectories similar to those of the four age periods i.e. a lagging period during childhood followed by a recuperation period from adolescence until adulthood (Figure S1), whereas Global IQ displayed again a stable deficit. (DOC) [file pone.0019153.s001.doc]

**SUPPORTING INFORMATION S1**

This information suggests that the results presented in the main text did not depend on the chosen developmental age cut-offs. To allow the construction of a developmental trajectory, the offspring sample was divided into subsamples of different age. Since there is no empirical evidence on which to base the choice of age cut-offs, we chose cut-offs that correspond to developmental ages that may have a meaning in social, clinical and developmental psychopathology. Thus, in the text of our article, a four age-period was chosen, as follow: primary school years (age 7-12), young adolescence (13-16), late adolescence (17-19) and beginning of adulthood (20-22)

When we re-examined the developmental patterns with three age periods (ages 7-14, 15-18, 19-22), the results remained congruent (**Table S1**).

To further analyze the three period intervals, it can be noted that the *Group* variable was significant for all the cognitive functions tested. As in the “four period analysis”, there were no statistically significant *Group x Age Periods* interaction terms for the Global IQ and for the verbal memory tests. In contrast, for the two tests of visual memory, the interaction term showed a statistical trend (RCFT_IR_, *p*=.071; RCFT_DR_, *p*=.099; **Table S1**) with shapes of the trajectories similar to those of the four age periods i.e. a lagging period during childhood followed by a recuperation period from adolescence until adulthood (**Figure S1**), whereas Global IQ displayed again a stable deficit.

**Table S1: Comparisons of offspring to controls in the total samples and in subsamples using three different age periods**

| Neuropsychological Variables | | | | | | | | | | | | | | | | | | | | | | | | |
| --- | --- | --- | --- | --- | --- | --- | --- | --- | --- | --- | --- | --- | --- | --- | --- | --- | --- | --- | --- | --- | --- | --- | --- | --- |
|  | Global IQ | | | |  | CVLT_TOT_ | | | |  | CVLT_DR_ | | | |  | RCFT_IR_ | | | |  | RCFT_DR_ | | | |
| Source | *df* | F | *p* | ES  *(CI)* |  | *df* | F | *p* | ES  *(CI)* |  | *df* | F | *p* | ES  *(CI)* |  | *df* | F | *p* | ES  *(CI)* |  | *df* | F | *p* | ES  *(CI)* |
| Groups^1^ | 1 | 23.51 | <.0001 | -0.81  *(-1.15, -0.46)* |  | 1 | 22.68 | <.0001 | -0.80  *(-1.15, -0.45)* |  | 1 | 30.70 | <.0001 | -0.93  *(-1.28, -0.58)* |  | 1 | 203.46 | <.0001 |  |  | 1 | 24.31 | <.0001 |  |
| Age periods | 2 | 2.29 | 0.10 |  |  | 2 | 4.75 | 0.01 |  |  | 2 | 3.45 | 0.03 |  |  | 2 | 9.80 | 0.0001 |  |  | 2 | 9.24 | 0.0002 |  |
| Groups X Age periods | 2 | 0.50 | 0.61 |  |  | 2 | 0.32 | 0.72 |  |  | 2 | 0.49 | 0.61 |  |  | 2 | 2.71 | 0.07 |  |  | 2 | 2.36 | 0.099 |  |
| Simple main effects |  |  |  |  |  |  |  |  |  |  |  |  |  |  |  |  |  |  |  |  |  |  |  |  |
| Offspring vs Controls at 7 - 14 years |  |  |  |  |  |  |  |  |  |  |  |  |  |  |  |  | -2.59 | 0.01 | -0.84  *(-1.49, 0.2)* |  |  | -2.12 | 0.04 | -0.69  *(-1.33, -0.04)* |
| Offspring vs Controls at 15 - 18 years |  |  |  |  |  |  |  |  |  |  |  |  |  |  |  |  | -4.29 | <.0001 | -1.39  *(-2.05, -0.73)* |  |  | -4.55 | <.0001 | -1.47  *(-2.13, -0.81)* |
| Offspring vs Controls at 19 - 22 years |  |  |  |  |  |  |  |  |  |  |  |  |  |  |  |  | -1.77 | 0.08 | -0.44  *(-0.93, -0.05)* |  |  | -2.49 | 0.01 | -0.62  *(-1.11, -0.13)* |

^1^ Comparison of total sample of offspring to total sample of controls

To verify the possibility that the results might depend on the chosen developmental age cut-offs, the developmental patterns was re-examined with three age periods ( ages 7-14, 15-18, 19-22).

*Non-independence of observations:* The 65 offspring sample was composed of 26 singletons and 17 sibships: 12 comprised two subjects and 5 comprised three. To account for possible correlation among subjects within the same sibship, a multilevel model was carried out using the MIXED procedure of SAS (version 9.2; SAS Institute Inc., Cary, NC). Sibships nested in the group were used as the second level and modeled according to a random effect. Degrees of freedom were obtained by the method of Kenward-Roger, 1997 [46] that is available with the option DDFM=KR in the MODEL statement of the MIXED procedure.

*Effect sizes (ES):* ES were calculated using the difference of adjusted means (LSMeans) between the experimental and control groups standardized by a pooled standard deviation. The pooled standard deviation was obtained by dividing the standard error of the difference of LSMeans by the square root of $\frac{1}{n_{1}}+\frac{1}{n_{2}}$, according to Kelley [47]. Confidence intervals (CI) for the effect sizes were obtained using the non-centrality interval estimation approach based on a *t* distribution, according to Steiger and Fouladi, 1997 [48]. The lower and upper bounds of the 95% CI were calculated by multiplying the 2.5% and 97.5% percentiles, respectively, of the non central *t* distribution by the square root of $\frac{n_{1}+n_{2}}{n_{1}\times n_{2}}$.

*–Interaction term:* A statistical trend for interaction term (*Group x Age Periods*), as described in *Statistical Analysis,* was found only for the two *Rey Complex Figures* Tests (immediate recall and delayed recall, respectively RCFT_IR_ and RCFT_DR_) suggesting that the cognitive differences for this domain varied with age. This imposed to interpret the effect sizes at each age period rather than the marginal one.

**Figure S1: Cross-sectional developmental trajectories representing the evolution in time of the IQ and memory impairments from 7 to 22 years of age, using three different age periods**

The developmental pattern for Global IQ (A), Visual episodic memory (B) and Verbal episodic memory (C) are illustrated for the controls (red line) and young offspring at genetic risk (blue line). The effect sizes (ES) express the magnitude of the difference between offspring and controls at each age period. A statistical trend for *Group x Age Periods* interaction terms was obtained only for the two tests of visual episodic memory (*p* = .071 for Rey Complex Figure Test immediate recall, RCFT_IR_, and *p* = .099 for Rey Complex Figure Test immediate recall, RCFT_DR_), which suggested that the cognitive differences varied with age for this function.
